# Supplementary material for: A miR-150/TET3 pathway regulates the generation of mouse and human non-classical monocyte subset
Source: Nat Commun. 2018 Dec 21;9:5455. doi: 10.1038/s41467-018-07801-x (PMC6303340; doi:10.1038/s41467-018-07801-x)
Supplement: Supplementary file 1 — Supplementary Information [file 41467_2018_7801_MOESM1_ESM.pdf]

Supplementary informations

A miR-150 / TET3 pathway regulates the generation of mouse and human non-classical monocyte subset

Selimoglu-Buet D. et al.

Selimoglu-Buet D et al, Supplementary Figure 1
(related to Figure 1)

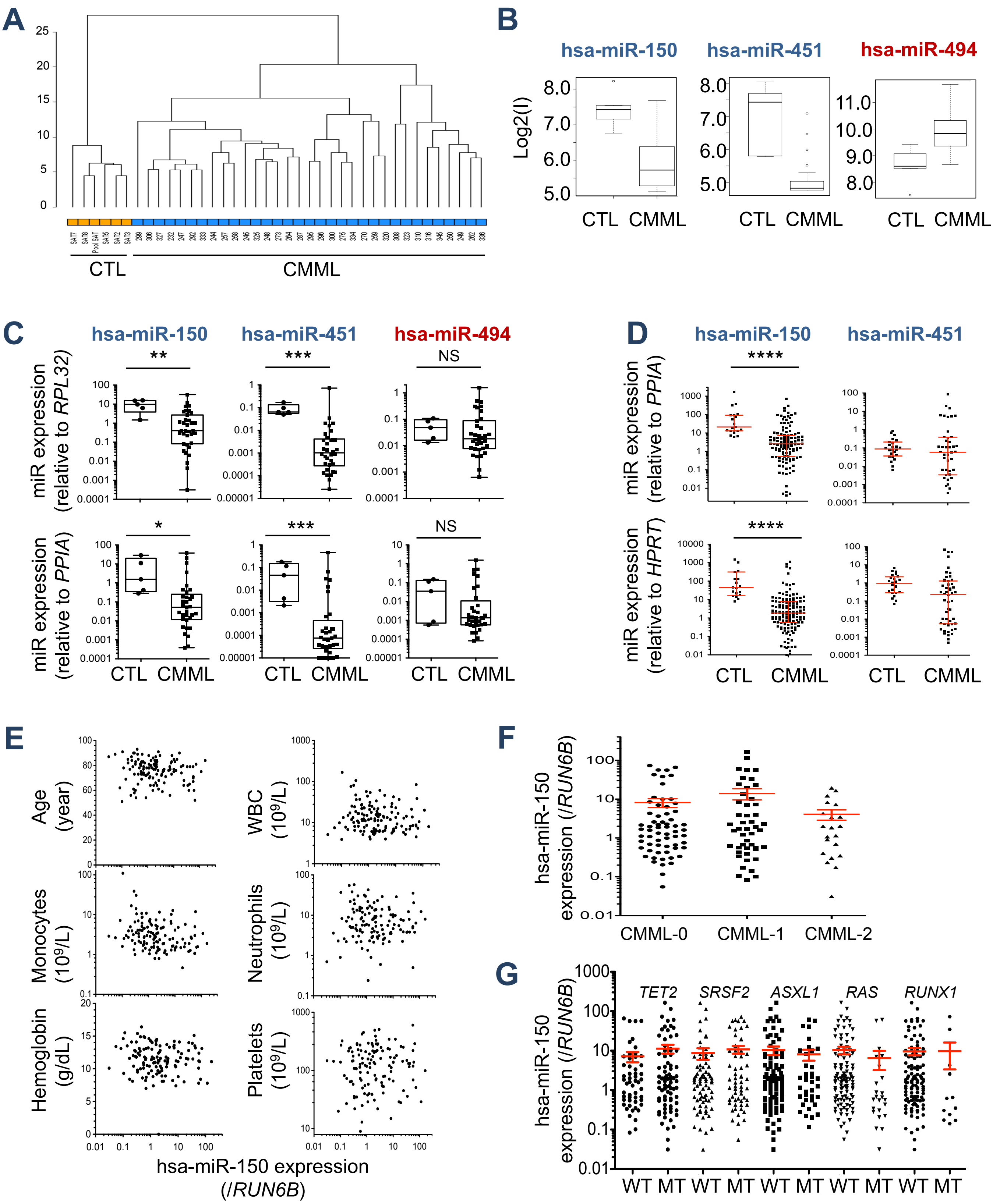

Supplementary Figure 1. Differential miRNA expression profiles in healthy controls or CMML sorted peripheral blood monocytes and relationship with clinical and biological features.

A. Hierarchical clustering of miRNA profiles obtained by microarray analysis (learning cohort), established using the Euclidean distance method and the Ward clustering method, and validated by boostraping (package pvclust). **B.** Box plot showing the variation in hsa-miR-150, hsa-miR-451 and hsa-miR-494 expression in samples of the learning cohort analyzed in figure 1A (centre line : median, whiskers: min to max). **C.** Box plot showing the relative expression of hsa-miR-150, hsa-miR-451 and hsa-miR-494 as measured by qRT-PCR in $CD14^+$ cell samples used for microarray analysis (learning cohort). Normalized to *L32* or *PPIA* (CTL=5; CMML=33), centre line : median, whiskers: min to max. **D.** qRT-PCR analysis of hsa-miR-150 and hsa-miR-451 expression in healthy donor and CMML patient $CD14^+$ cells of controls and CMML patients (validation cohort). hsa-miR-150 normalized to *PPIA* (CTL=19; CMML=124) or *HPRT* (CTL=19; CMML=143). hsa-miR-451 normalized to *PPIA* (CTL=24; CMML=44) or *HPRT* (CTL=24; CMML=52). Red lines: median with interquartile range. Mann-Whitney test: * P<0.05; ** P<0.01; *** P<0.001; **** P<0.0001. **E-G.** Relationship between hsa-miR-150 expression and CMML clinical and biological features. hsa-miR-150 expression relative to *RNU6B* was quantified in the validation cohort of 139 CMML patient $CD14^+$ monocytes and correlated to disease features such as age, white blood cell count (WBC), monocyte count, neutrophil count, hemoglobin level (Hb), platelet count (**E**), or WHO 2016 diagnosis criteria (CMML-0, -1, -2) (**F**), and mutational status for *TET2, SRSF2, ASXL-1, RAS (NRAS and KRAS), RUNX*-1; WT: not mutated, MUT: mutated, (**G**). Red lines: mean +/-SEM.

Selimoglu-Buet *et al.*, Supplementary Figure S2 *(related to Figure 2)*

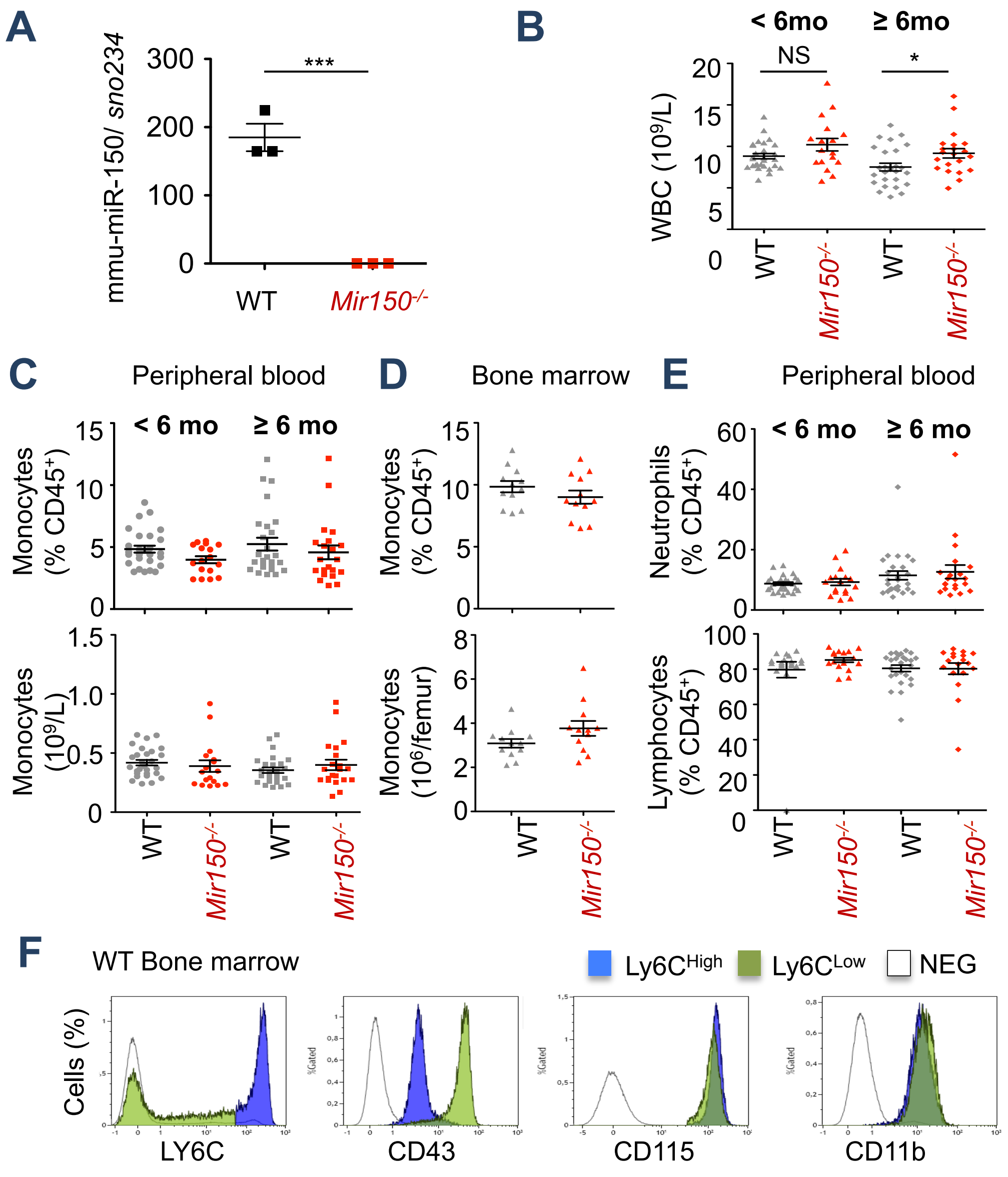

Supplementary Figure 2. Hematopoietic cell populations in miR-150$^{-/-}$ mice.

A. mmu-miR-150 expression was measured by qRT-PCR in bone marrow cells of 3 *Mir150$^{-/-}$* (KO) and 3 wildtype (WT) mice and normalized to *sno234*. Mean +/-SEM, unpaired t test, *** P<0.001. **B.** White blood cell count in the peripheral blood of young (< 6 months, WT=28; KO=17) and older (≥ 6 months, 6 to 26 months; WT=26; KO=21) mice. Mean +/SEM, unpaired t test, NS, non significant, * P<0.05. **C.** Flow quantification of monocytes in the peripheral blood (percentage of CD45^{+} cells or absolute number) of young and older mice, as in B. **D**. Flow quantification of monocytes in bone marrow (percentage of CD45^{+} cells or number /femur). N=12 mice /group. **E.** Flow quantification of neutrophils and lymphoid cells in the peripheral blood of young and older mice, as in B. Mean +/SEM, unpaired t test, non significant for all panels presented. **F.** Representative analysis of cell surface marker expression on bone marrow monocyte subsets of WT mice by flow cytometry (green: Ly6C^{low} cells, blue: Ly6C^{high} cells, white: negative cells).

Selimoglu-Buet D *et al.*, Supplementary Figure 3 *(related to Figure 2)* Part 1

A Exclusion gating strategy

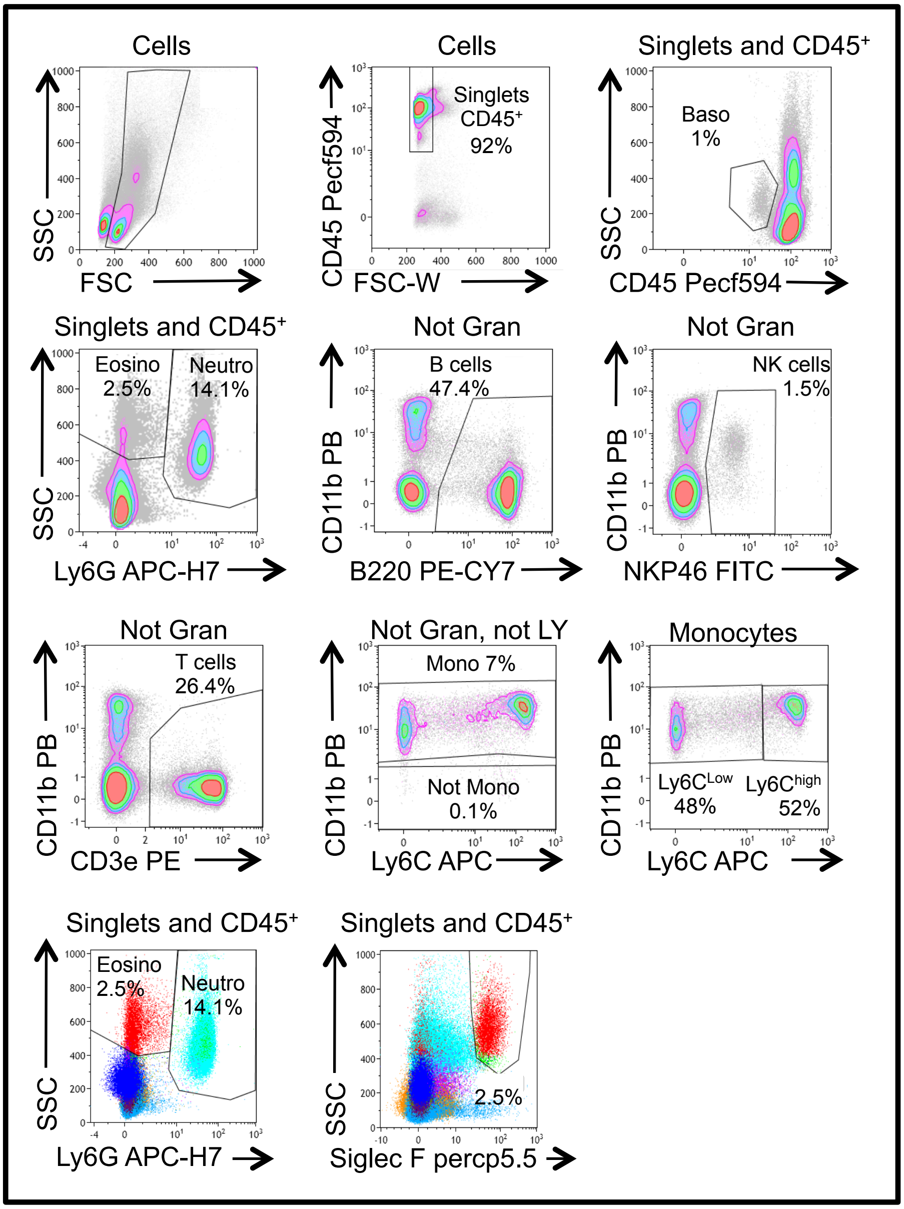

Selimoglu-Buet D *et al.*, Supplementary Figure 3 *(related to Figure 2)* Part 2

B CD115^{+} gating strategy

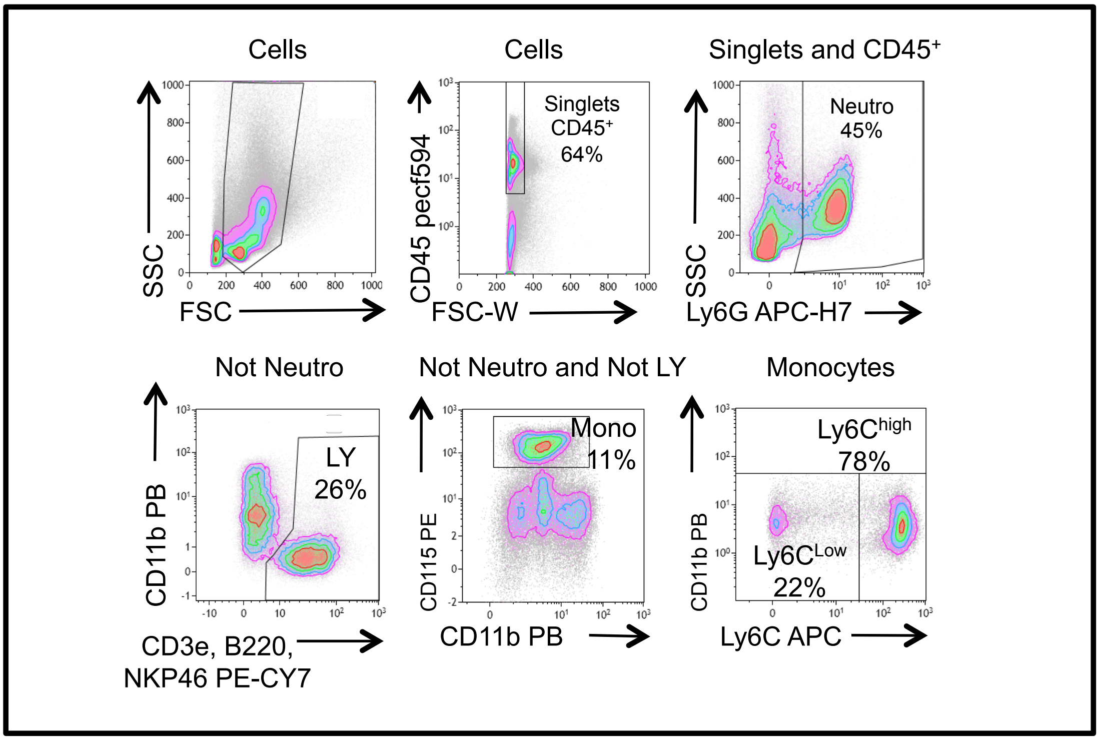

C Comparison of the two gating strategies

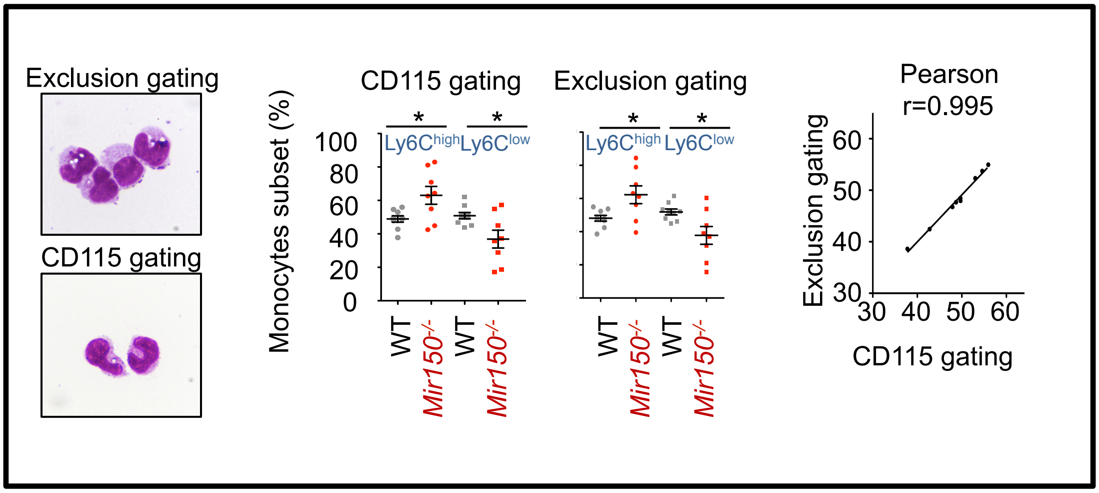

Supplementary Figure 3. Flow cytometry quantification of monocyte subsets.

A. Exclusion gating strategy, used for blood analysis (Figures 2D,E,F; 3A,B,D,E,F,H; 8E,F; Supplementary Figures 2C,E; 3A,B,C,D; 4D; 10F): from total cells, $CD45^+$ and singlets were selected. CD45.1 and CD45.2 staining was used to distinguish donor and receiver hematopoietic cells. Then, basophils ($CD45^{low}$,SSC^{low}), neutrophils ($Ly6G^+$) and eosinophils ($CD45^+$,SSC^{high}) were excluded. In the so-defined "Non-granulocytes" gate, $B220^+$ B cells, $CD3^+$ T and $NKP46^+$ NK cells were excluded**.** Monocytes were gated in remaining cells as $CD11b^+$ cells and divided into $Ly6C^{high}$ and $Ly6C^{low}$ subsets. Eosinophil gating ($CD45^+$,SSC^{high}) was validated by Siglec F staining (eosinophils in red). Percentages of cells among $CD45^+$ and Singlets are shown. **B**. CD115-based selection used for bone marrow analysis (Figures 2A,B,C; Supplementary Figures 2D,F): from total cells, $CD45^+$ and singlets were selected. $Ly6G^+$ neutrophils were excluded, then $CD3^+$, $B220^+$ and $NKP46^+$ cells were excluded. Monocytes were selected in remaining cells as $CD115^+$ and $CD11b^+$ expressing cells, which were subsequently divided into $Ly6C^{high}$ and $Ly6C^{low}$ subsets. Percentages of cells among "$CD45^+$ and Singlets" are shown. **C.** Monocytes from WT mice peripheral blood gated through these 2 strategies were sorted and stained with May-Grünwald-Giemsa (left panel, representative of 3 experiments), the fraction of $Ly6C^{high}$ and $Ly6C^{low}$ subsets was quantified using these 2 strategies in the peripheral blood of wildtype (WT) and *Mir150*$^{-/-}$ (KO) mice (9 in each group) (mean +/-SEM, unpaired t test, middle panel) and correlation between $Ly6C^{high}$ fraction measured by each method in WT mice was examined (Pearson r^2= 0.9901, $P<0.0001$, equation, Y=0.98X) (Right panel).

Selimoglu-Buet D et al., Supplementary Figure 4
(related to Figure 2)

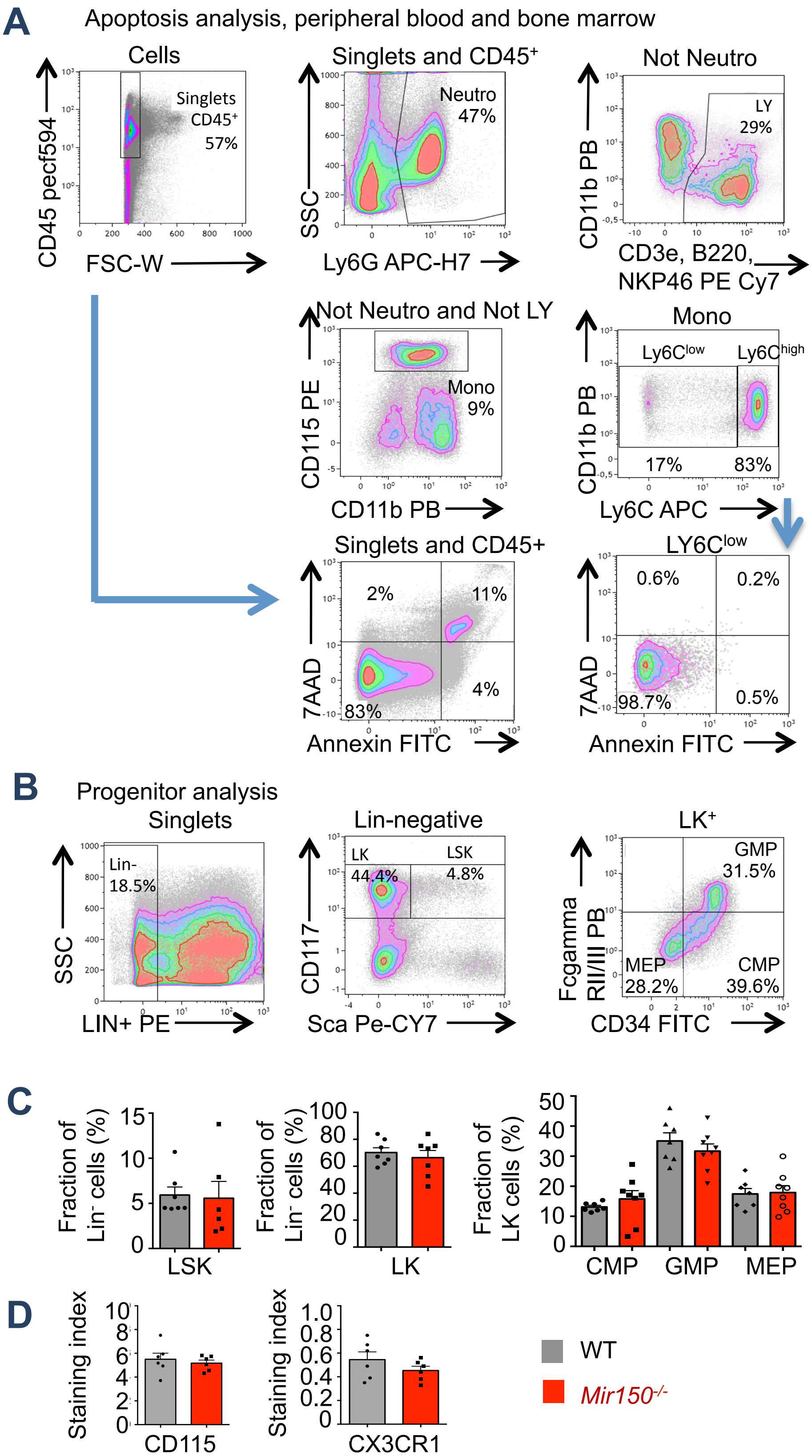

Supplementary Figure 4. Flow cytometry quantification of apoptotic cells and progenitors in *Mir150$^{-/-}$* mice. A. Gating strategy for apoptosis quantification in bone marrow samples (Figure 2G) started with the selection of CD45^{+} and singlets cells prior to gating on Ly6C^{high} and Ly6C^{low} monocytes as in figure S3B. Same gating strategy was used for blood samples. Lower panels provide examples of early (Annexin V^{+}, 7AAD^{-}) and late (Annexin V^{+}, 7AAD^{+}) apoptosis detection in singlets, CD45^{+} (left) and Ly6C^{low} (right) bone marrow cells collected from a *Mir150$^{-/-}$* animal. **B.** Flow quantification of mouse bone marrow progenitors (Supplementary Figure 4C): from singlets, lineage negative (B220^{-}, CD3^{-}, CD19^{-}, GR1^{-}, Ter119^{-}, NK1.1^{-}, LY6G^{-}, LY6C^{-}) were excluded; of Lin^{-} cells, LSK (CD117^{+}Sca-1^{+}) and LK (CD117^{+}Sca-1^{-}) population were selected. LK cells were further subdivided into CMP (CD34^{+}), GMP (CD34^{+}FcgammaRII/III^{+}) and MEP (CD34^{-} FcgammaRII/III^{-}). **C.** Fraction of LSK and LK among Lin^{-} cells, and fraction of CMP, GMP, MEP among LK cells, in wildtype (WT, grey) and *Mir150$^{-/-}$* (red) mice. Mean +/-SEM, N= 7 mice per group. **D.** CD115 and CX3CR1 expression by flow cytometry on Ly6C^{low} monocytes of wildtype (WT, grey) and *Mir150$^{-/-}$* (KO, red) mice. Staining index, Mean +/-SEM, N= 6 mice per group.

Selimoglu-Buet D *et al.*, Supplementary Figure 5
(related to Figure 3)

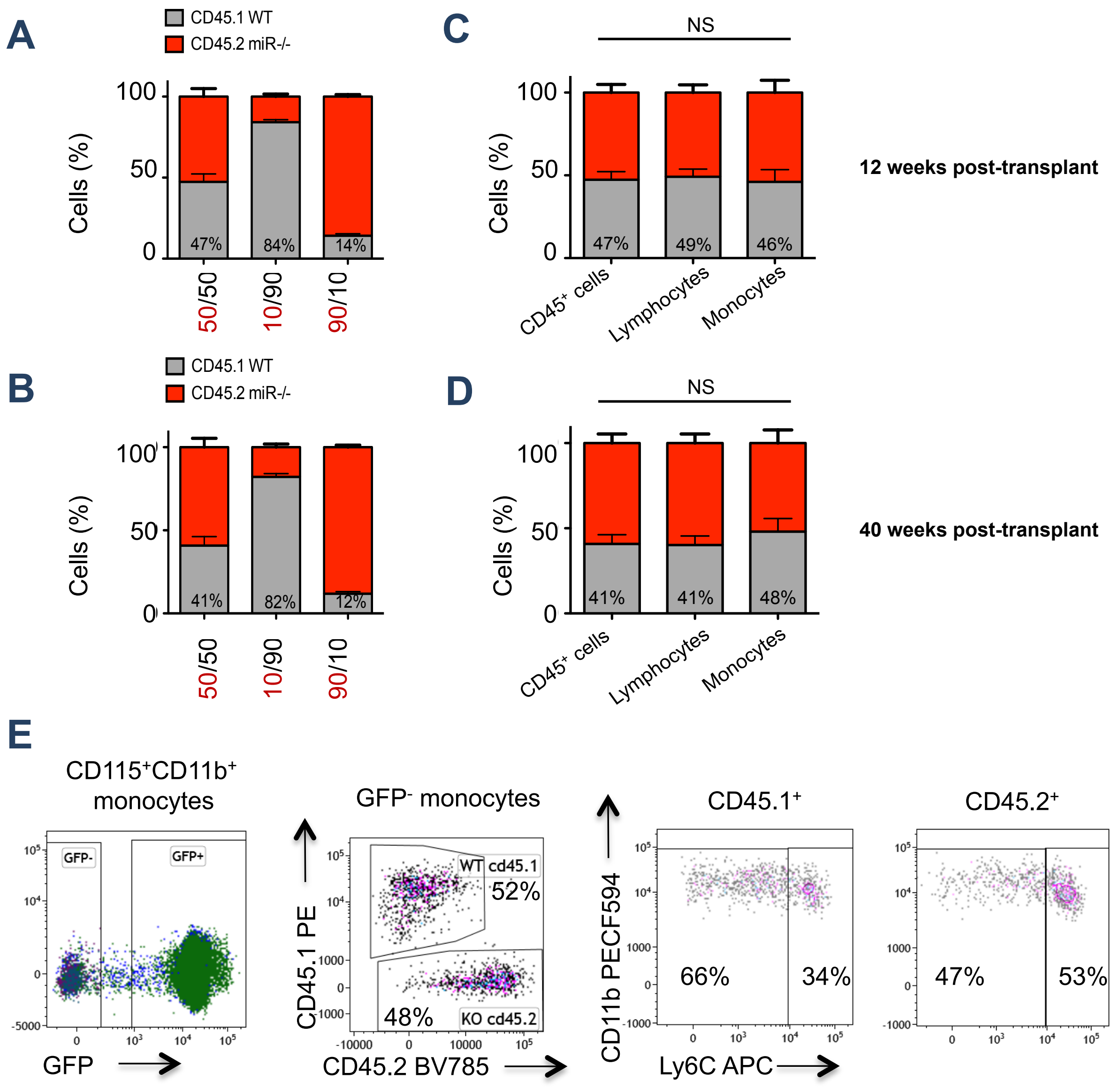

Supplementary Figure 5. Cell-autonomous function of miR-150 in mouse monocyte subset generation. **A, B.** Fraction of CD45.1^{+} (WT, grey) and CD45.2^{+} (KO, red) cells among CD45^{+} peripheral blood cells analyzed 12 (**A**) and 40 (**B)** weeks after competitive transplantation at indicated ratio (N=6 per group, mean +/-SEM). **C, D.** Expression of CD45.1 and CD45.2 in indicated cell subsets measured 12 (**C**) and 40 (**D)** weeks after competitive transplantation at a 50/50 ratio (N=6 per group, mean +/-SEM). **E.** Gating strategy for monocyte transfer analysis (presented Figure 3J). Dead cells, Ly6G^{+}, CD3^{+}, B220^{+} and NK1.1^{+} cells were excluded and monocytes were selected as CD115^{+} and CD11b^{+} expressing cells (as presented Supplementary Figure 3B). Transferred monocytes were selected as GFP^{-} cells, separated on CD45.1 or CD45.2 expression and subsequently divided into Ly6C^{high} and Ly6C^{low} subsets.

Selimoglu-Buet D et al., Supplementary Figure 6
(related to Figure 4)

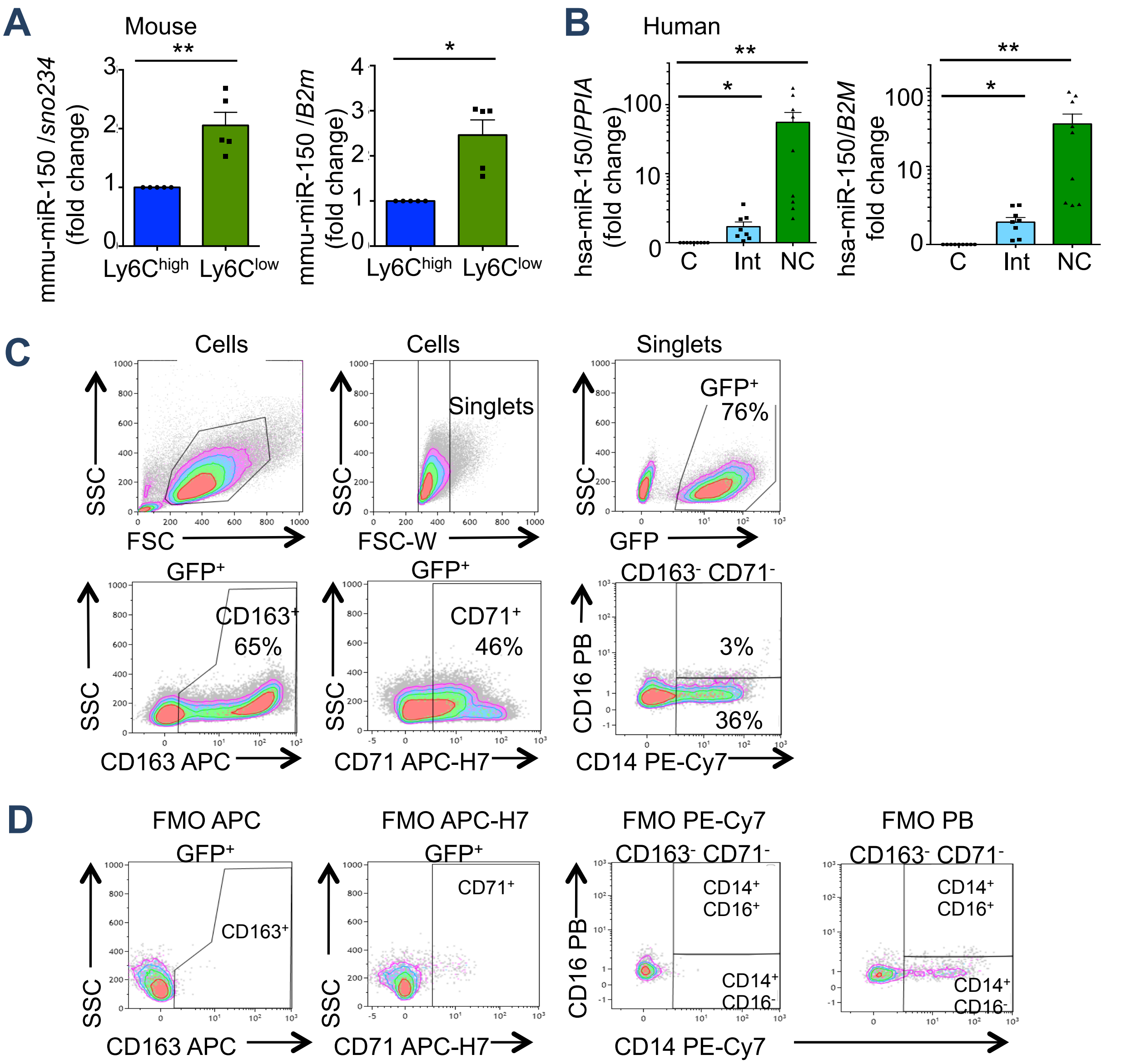

Supplementary Figure 6. miR-150 is differentially expressed in mouse and human monocyte subsets. A. qRT-PCR analysis of the expression of mmu-miR-150 in sorted Ly6C^{high} and Ly6C^{low} monocyte subsets from wildtype mice, normalized by *B2M* or *sno234*. Results are expressed as mmu-miR-150 expression fold change between Ly6C^{high} (normalized to 1) and Ly6C^{low}. Mean +/-SEM of 5 mice, Paired t test, * P<0.05, **P<0.01. **B.** qRT-PCR analysis of the expression of hsa-miR-150 in classical (C), intermediate (Int) and nonclassical (NC) monocyte subsets from 9 healthy donors sorted by flow cytometry. hsa-miR-150 expression is normalized to *HPRT* or *PPIA* expression. Results are expressed as fold change between classical (normalized to 1) and intermediate or nonclassical monocytes. Mean +/-SEM, paired t test * P<0.05, **P<0.01. **C.** Gating strategy for monocytes quantification in differentiation assay (Figure 4C,D,E). From singlets cells, transduced cells were selected on GFP. Among GFP^{+} cells, macrophages were excluded, based on CD163 and CD71 expression. Monocytes were divided in two subsets based on the expression of CD14 and CD16. **D.** Positive staining for CD163, CD71, CD14 or CD16 was delimited on the basis of fluorescence minus one (FMO) sample for each antibody.

Selimoglu-Buet D et al., Supplementary Figure 7
(related to Figure 5)

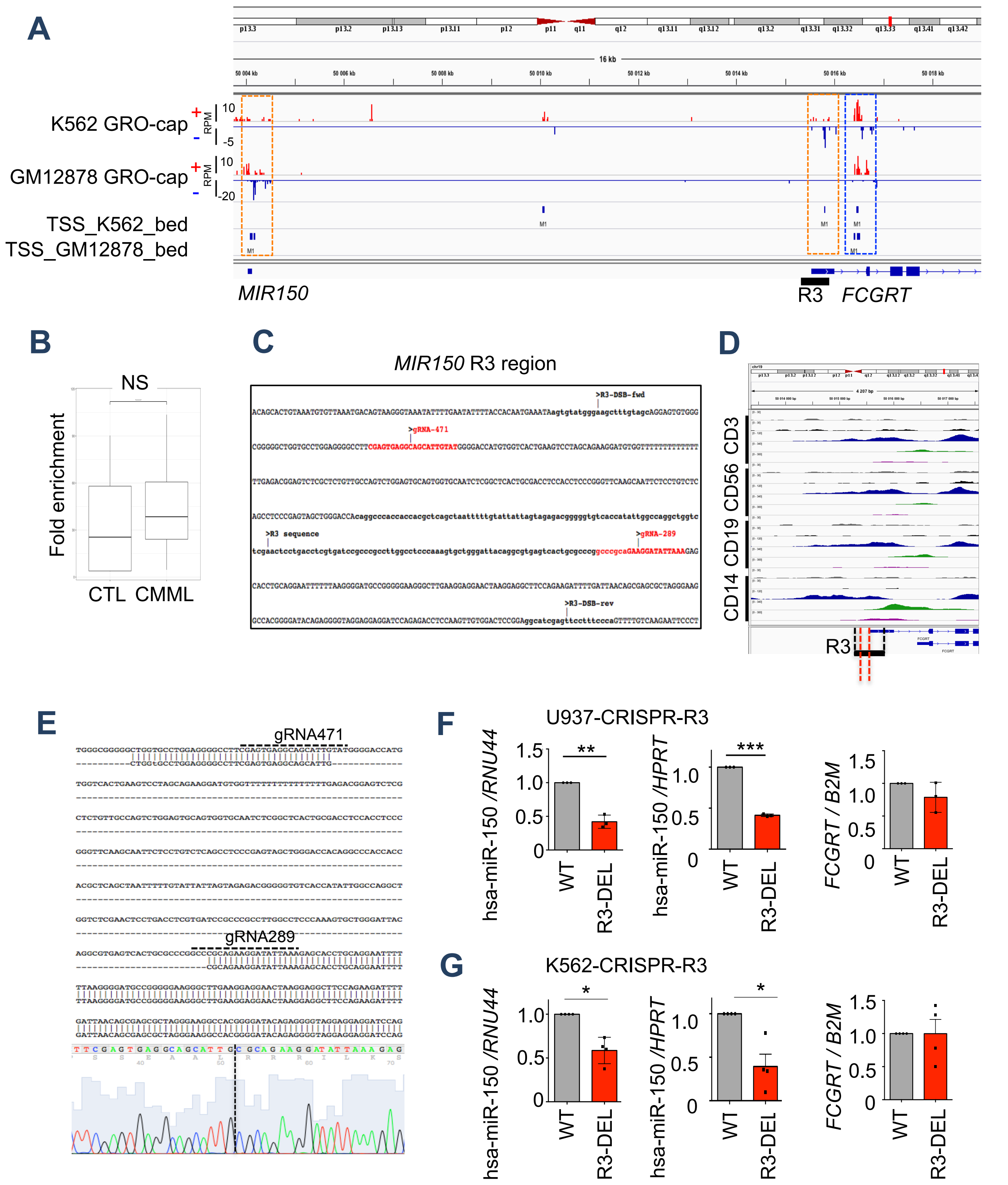

Supplementary Figure 7. CRISPR/Cas9-induced deletion of R3 region.

A. GRO-cap analysis of TSSs in a human myeloid (K562) and a human lymphoid (GM12878) cell line. IGV shot of *MIR150* / *FCGRT* locus. Red, plus strand; blue, minus strand. The bed files of all TSS found in K562 and GM12878 are shown. R3 region is shown. **B.** Fold enrichment of H3K4Me3 peaks in R3 region (chr19: 50,015,000-50,018,000) in $CD14^{+}$ monocytes of healthy donors (CTL, N=3) and CMML patients (N=4) (Wilcoxon signed-rank test, p-value = 0.609, NS not significant). Fold enrichment distribution displayed as a boxplot (centre line : median, whiskers: min to max). **C.** Partial DNA sequence of *MIR150* region R3 (represented in lower cases) and surrounding regions with CRISPR/Cas9 guides RNA shown in red. **D.** Snapshot of Figure 5C zooming on R3 region mapping the CRISPR/Cas9-deleted sequence in red. **E**. Sanger sequencing of a homozygous clone. Top panel: alignment of sequences shows the partial deletion of R3 region between the two guide RNAs. Bottom panel: visualization with 4Peaks sequence viewer showing the perfect junction between the two cuts on the sequence trace. **E, F.** Expression of hsa-miR-150 or *FCGRT* in U937 (**E**) or K562 (**F**) clones in which *MIR150* R3 has been partially deleted. Results are expressed as expression fold change between wildtype clone (WT, =1, grey bars) and R3-deleted clone (R3-DEL, Red bars). has-miR-150 expression is normalized to that of *RNU44* or *HPRT. FCGRT* expression is normalized to *B2M* (similar results with *RPL32* or *HPRT* as normalizers). Results are mean +/-SD of a minimum of 3 independent experiments. Paired t test, * P<0.05; ** P<0.01; *** P<0.001.

Selimoglu-Buet D et al, Supplementary Figure 8
(related to Figure 6)

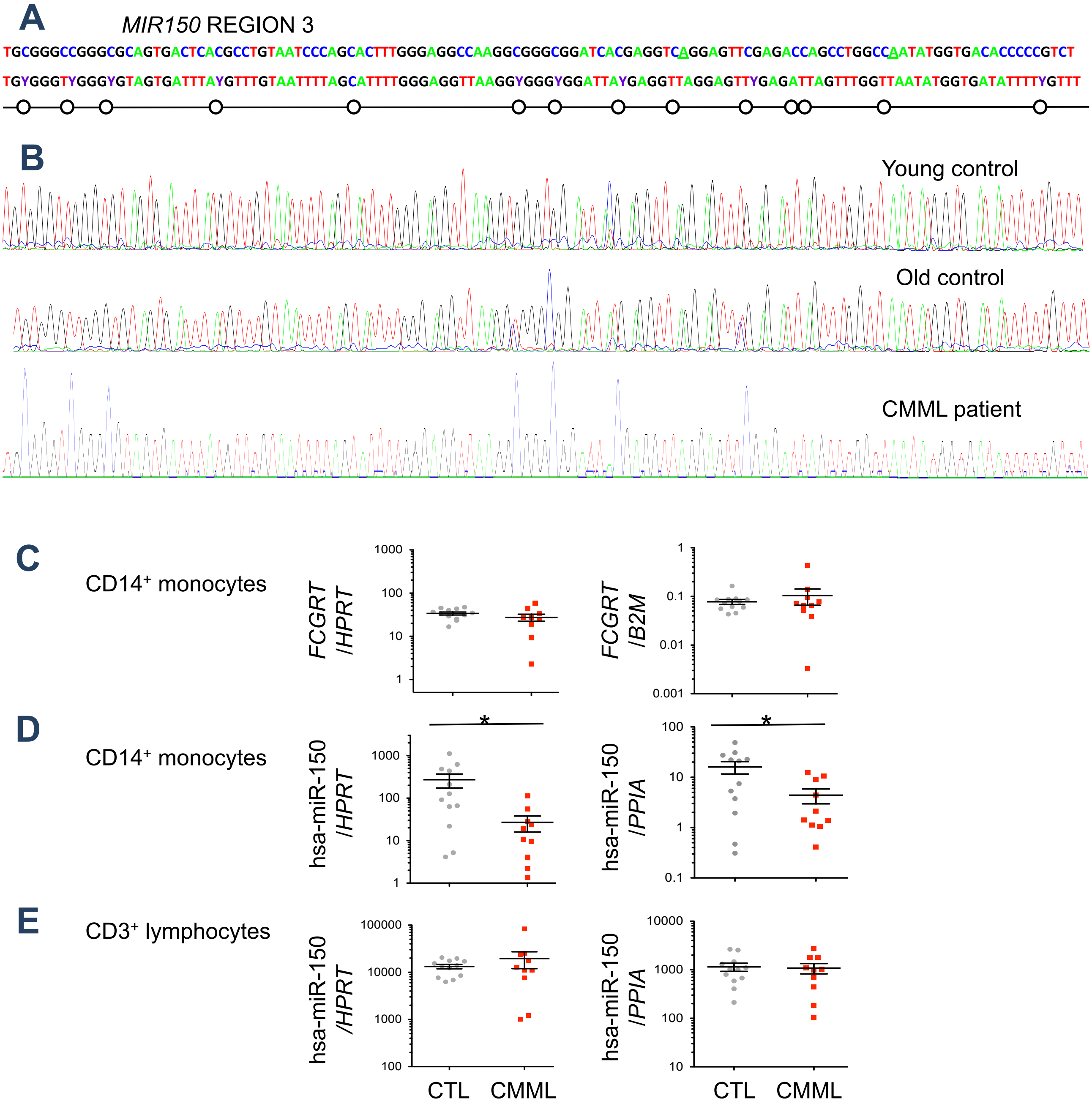

Supplementary Figure 8. DNA methylation status of *MIR150* region three (R3) and expression of hsa-miR-150 in cells from CMML or controls. A. DNA sequence of analyzed R3 (upper line), bisulfite-converted sequence (middle line), and schematic representation of putative methylated sites. **B**. Example of DNA sequencing of R3 region after bisulfite conversion in monocytes from a young healthy donor (Young control) an older healthy donor (Old control) and a CMML patient. **C, D, E.** Expression level of *FCGRT* and hsa-miR-150 in healthy donor and CMML patients. Samples are those studied by bisulfite sequencing, healthy donors (CTL, N=12) and CMML patients (N=10). **C.** qRT-PCR analysis of *FCGRT* gene expression in CD14$^+$ monocytes, normalized to *HPRT* (left) or *B2M* (right). **D**,**E**. qRT-PCR analysis of hsa-miR-150 expression in CD14$^+$ monocytes (**D**) and in CD3$^+$ T lymphocytes (**E)**, normalized to *HPRT* (left) or *PPIA* (right). Mean +/-SEM, unpaired t test, * P<0.05.

Selimoglu-Buet D et al., Supplementary Figure 9 (related to Figure 7)

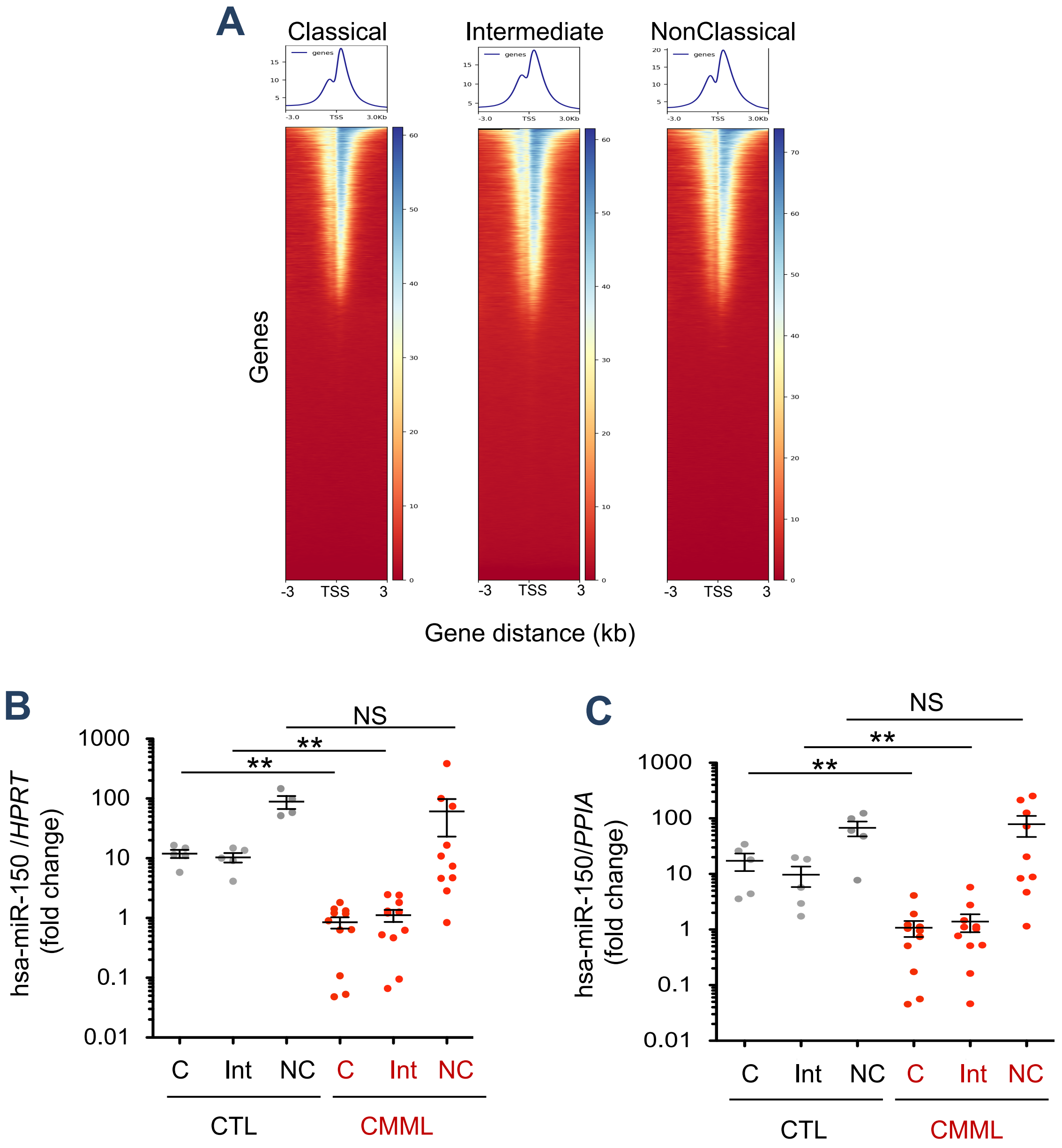

Supplementary Figure 9. DNA methylation status of *MIR150* promoter region three (R3) and expression of hsa-miR-150 in cells in human monocyte subsets. A. Representative H3K4me3 ranking heat maps centered on gene transcription starting site (TSS) of sorted classical, intermediate and nonclassical monocytes collected from one healthy donor. **B, C.** qRT-PCR analysis of hsa-miR-150 expression normalized to *HPRT* (**B**) or *PPIA* (**C**) in classical (C), intermediate (Int) and nonclassical (NC) monocytes sorted from peripheral blood samples of 8 healthy donor controls (CTL) and 12 CMML patients. Mean +/-SEM; Mann-Whitney test: ** P<0.01; ***P<0.01, NS non significant.

Selimoglu-Buet D et al., Supplementary Figure 10
(related to Figure 8)

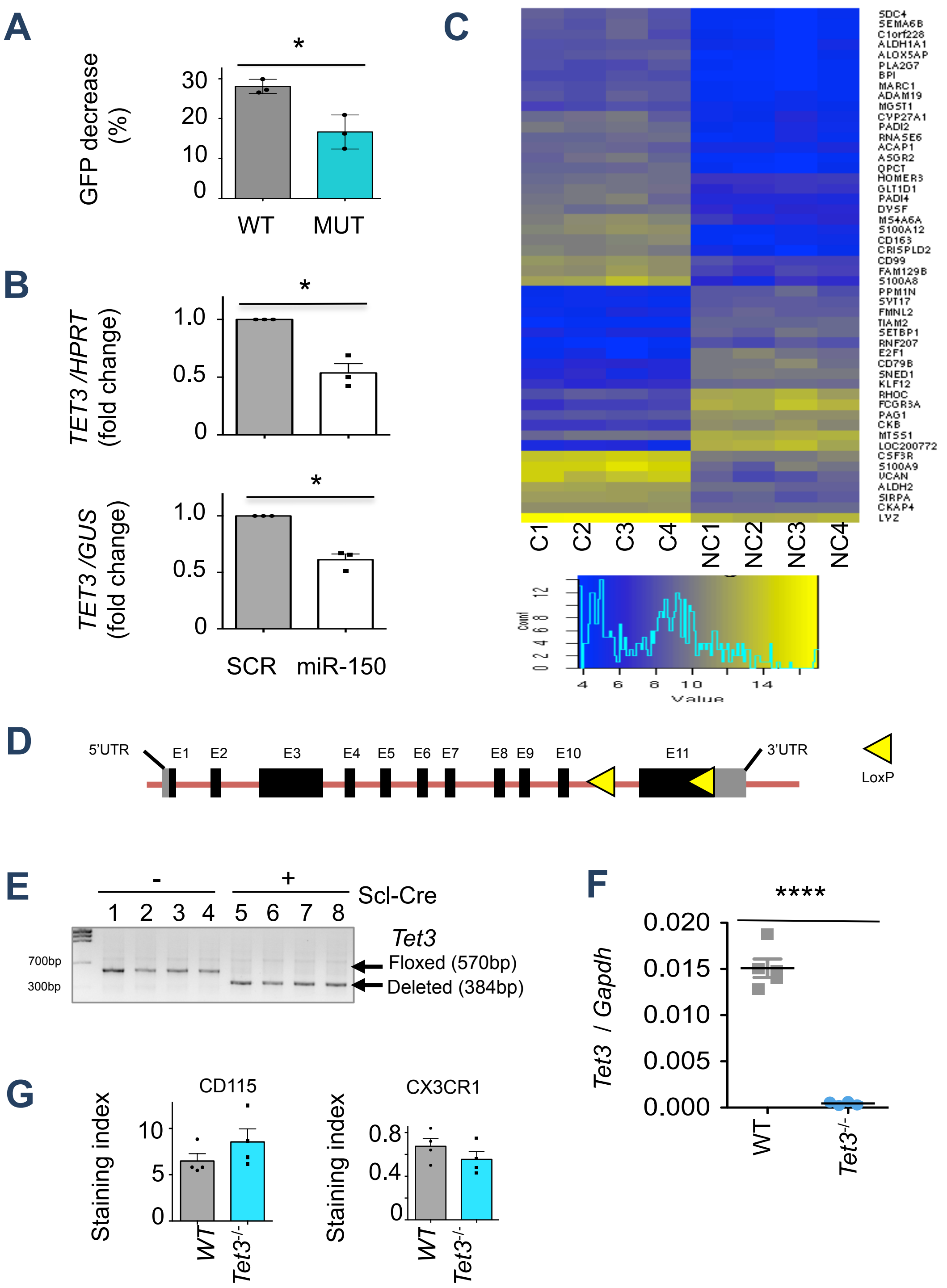

Supplementary Figure 10. *TET3* is a miR-150 target involved in monocyte subset differentiation. **A.** Validation of *TET3* as a miR-150 target in U937 cell line. A GFP reporter assay was used, with (MUT) and without (WT) mutation of predicted interaction sites of miR-150 with *TET3* 3'-UTR. The decreased fraction of GFP measured 48 hours after lentiviral transduction is shown (mean+/-SD, N=3 independent experiments. Paired t test, * P<0.05). **B***.* *TET3* expression relative to *HPRT* and *GUS* was quantified in monocytes generated by five days in culture of human $CD34^+$ cells transduced with hsa-miR-150-GFP compared to control (SCR-GFP). Results are expressed as fold change in *TET3* expression in miR-150-GFP relative to SCR-GFP expressing cells. Mean +/-SEM of 3 independent experiments. Paired t test, * P<0.05. **C.** Heat map showing the expression of the 50 most discriminating genes between classical and nonclassical human monocyte subsets analyzed by RNA sequencing of four healthy donors. **D.** Genomic organization of *Tet3* gene. LoxP sites were introduced into intron 10 and the 3'UTR of exon 11. E, exon; **E**. Multiplex PCR analyses of Scl-Cre-mediated deletion of *Tet3*-floxed alleles in sorted monocytes. Both Cre- (1, 2, 3 and 4) and Cre+ (5, 6, 7 and 8) two month-old mice were treated with tamoxifen. PCR shown here are done 7-months (3, 4, 7 and 8) and 16-months (1, 2, 5 and 6) after last tamoxifen treatment. **F**. Quantitative real-time PCR analysis of *Tet3* transcript level in sorted monocytes from control (N=5) and $Tet3^{-/-}$ mice (n=4). Results normalized to *Gapdh*, unpaired t test ****P < 0.0001. **G.** CD115 and CX3CR1 expression by flow cytometry on $Ly6C^{low}$ monocytes collected from wildtype (WT, grey) and $Tet3^{-/-}$ (blue) mice. Staining index, Mean +/-SEM of 4 mice per group.

Selimoglu-Buet D *et al.*, Supplementary Figure 11

TET3 uncropped scan, predicted size : 179 kDa

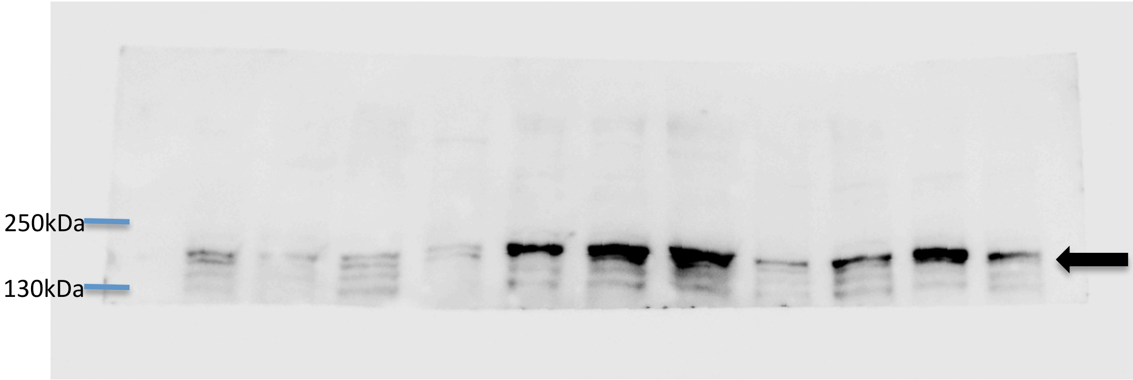

Adaptin uncropped scan, predicted size : 100 kDa

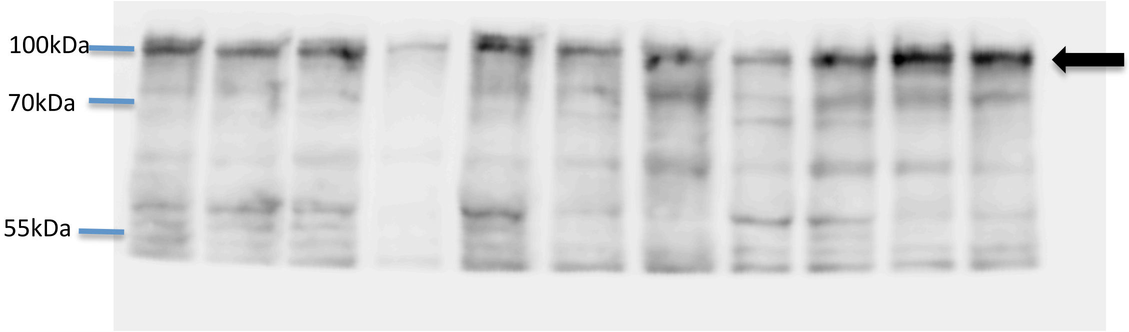

Supplementary Figure 11. Uncropped scans of TET3 and ADAPTIN immunoblots.

Supplementary Table 1. Characteristics of patients included in the learning and in the validation CMML cohorts.

Patient characteristics	Learning cohort n=33	Validation cohort n=139
Age years, median [range]	73 [56-88]	79 [51-93]
Male, n (%)	27 (82%)	94 (68%)
Platelets, x10^9 /L, median [range]	128 [12-560]	182 [9-605]
Hb, g/dL, median [range]	10.0 [7-14]	11.5 [7-16.4]
WBC, x 109 /L, median [range]	25.8 [4.1-81.7]	22.8 [3.1-167.4]
Neutrophils, x109 /L, median [range]	11.7 [0.4-40]	12.6 [0.24-57.2]
Monocytes, x109 /L, median [range]	8.2 [1.1-33.2]	6.3 [1-110]
Bood monocytes, %, median [range]	31.0 [5.1-65]	30.8 [10-77]
Marrow blasts, %, median [range]	8 [1-19]	7 [0-19]
Myelodysplasic form, n (%)	8 (24%)	73 (52.5%)
WHO 2016		
CMML-0, n (%)	6 (18%)	66 (47%)
CMML-1, n (%)	15 (45%)	48 (35%)
CMML-2, n (%)	12 (36%)	21 (15%)
ASXL1, mutated/ studied, %	16/ 31 (52%)	37/ 137 (27%)
TET2, mutated/ studied, %	17/ 33 (52%)	82/ 136 (60%)
RUNX1, mutated/ studied, %	10/ 33 (30%)	12/ 139 (9%)
RAS,* mutated/ studied, %	13/ 33 (39%)	25/ 139 (18%)
CBL, mutated/ studied, %	3/ 33 (9%)	17/ 139 (12%)
SRSF2, mutated/ studied, %	11/ 23 (48%)	56/ 133 (42%)
JAK2, mutated/ studied, %	1/ 33 (3%)	11/ 139 (8%)

* *RAS* mutated indicates mutation in *NRAS* or *KRAS* or *CBL* genes

Supplemental Table 5. Antibodies for Flow cytometry analyses and sorting

Antigen	Clone (Isotype)	Fluorochrome	Source	Catalog nb	Dilution
**Mouse antibodies**					
CD45	30-F11 (IgG2b, K)	PE-CF594	BD	562420	1/100
CD3e chain	145-2C11 (IgG1, K)	PE	BD	553063	1/100
CD45R/B220	RA3-6B2 (IgG2a, K)	PE-Cy7	BD	563103	1/200
NKp46, CD335	29A1.4 (IgG2a, K)	FITC	BD	560756	1/100
CD11b	M1/70 (IgG2b, K)	BV450	BD	560455	1/100
Ly6G	1A8 (IgG2a, K)	APC-H7	Biolegend	127624	1/100
Ly6C	HK1.4 (IgG2c, K)	APC	Biolegend	128016	1/500
CD43	S7 (IgG2a, K)	BV510	BD	563206	1/100
CD3e chain	145-2C11 (IgG1, K)	PE-Cy7	BD	561100	1/100
NKp46, CD335	29A1.4 (IgG2a, K)	PE-Cy7	Biolegend	137617	1/100
CD115	AFS98 (IgG2a, K)	biotin	Biolegend	135508	1/200
CD115	AFS98 (IgG2a, K)	BV605	Biolegend	135517	1/500
CD45.1	A20 (IgG2a, K)	PE-Cy7	Biolegend	110729	1/100
CD45.2	104 (IgG2a, K)	Percp5.5	BD	552950	1/100
CD11b	M1/70 (IgG2b, K)	PE-CF594	BD	562317	1/100
CD45.1	A20 (IgG2a, K)	PE	Biolegend	110707	1/100
CD45.2	104 (IgG2a, K)	BV785	Biolegend	109839	1/100
Siglec-F	E50-2440 (IgG2a, K)	Percp5.5	BD	565526	1/100
CD34	RAM34 (IgG2a, K)	FITC	BD	560238	1/100
CD117	2B8 (IgG2b, K)	APC	BD	561074	1/100
SCA	D7 (IgG2a, K)	PE-Cy7	BD	558162	1/100
CX3CR1	SA011F11	BV711	Biolegend	149031	1/100
CD16/32	2.4G2 (IgG2b, K)	Pacific Blue	BD	560540	1/100
Lin	(CD3e, CD11b, B220, TER119, Gr1)	biotin	Biolegend	133307	2uL each / 10^6cell
CD3e chain	145-2C11 (IgG1, K)	biotin	Biolegend	100304	0.25 uL/10^6cell
Ly6G	1A8 (IgG2a, K)	biotin	Biolegend	127604	0.25 uL/10^6cell
CD45R/B220	RA3-6B2 (IgG2a, K)	biotin	Biolegend	103204	0.25 uL/10^6cell
Ter119	Ter119	biotin	Biolegend	116203	0.25 uL/10^6cell
Nk1.1	PK136	biotin	Biolegend	108704	0.25 uL/10^6cell
**Human antibodies**					
CD163	GHI/61.1	APC	Miltenyi	130-100-612	1/100
CD71	YDJ1.2.2 (IgG1)	APC-H7	Beckman Coulter	A89313	1/100
CD14	RMO52 (IgG2a)	PE-CY7	Beckman Coulter	A22331	1/100
CD16	3G8 (IgG1)	Pacific Blue	Beckman Coulter	A82792	1/100
CD45	J.33 (IgG1)	Krome orange	Beckman Coulter	A96416	1/100
CD24	ALB9 (IgG1)	BUV395	BD	566221	1/100
CD14	M5E2 (IgG2a, K)	BV421	BD	563743	1/100
CD16	3G8 (IgG1, K)	APC-CY7	BD	560195	1/100
CD56	N901 (HLDA6 IgG1)	APC	Beckman Coulter	IM2474U	1/100
**Chemicals**					
Streptavidin		PE	BD	554061	
7AAD			Thermofisher	00-6993-50	
Annexin V		FITC	BD	556547	
Sytox		Pacific Blue	Thermofisher	S34857	

Supplementary Methods.

GFP reporter assay. Two hsa-miR-150 sites predicted by Targetscan (http://www.targetscan.org/) in the 3'UTR sequence of *TET3* were synthetized (Eurofins-Genomics) as wild-type ("WT1+WT2") or mutated ("MUT1+MUT2") sites as described in[1], separated by a small AATT sequence, as follow: Sal1-WT1+WT2-Xma1, F: 5'-TCGACTCCTCTCTGATAGGATGGGAGAGAATTTTTAACATGTAATTGTGGGAGAAC-3', R: 5'-CCGGGTTCTCCCACAATTACATGTTAAAAATTCTCTCCCATCCTATCAGAGAGGAG-3' and Sal1-MUT1+MUT2-Xma1, F: 5'-TCGACTCCTCTCTGATAGGATATAATAGAATTTTTAACATGTAATTGTATAATAAC-3', R: 5'-CCGGGTTATTATACAATTACATGTTAAAAATTCTATTATATCCTATCAGAGAGGAG-3'. Sequences were annealed and ligated with a XmaI/SalI digested sinpRRL-PGK-GFP reporter plasmid. miR-150 putative sites were cloned after the GFP cassette generating a GFP reporter assay. WT1-WT2 and MUT1-MUT2 constructions were validated by SANGER sequencing. The miR-150 expressing vector was constructed from the sinpRRL-hsa-mir150-PGK-GFP (described in Methods, section Plasmid constructs, viral production and titration). The PGK-GFP cassette was removed by EcoRV/SalI digestion and the extremities were blunted and ligated together. U937 cells (dilution: 10^6/ mL) were transduced with particles encoding the GFP reporter constructs (MOI=5) associated or not with particles encoding miR-150 (MOI=20). GFP expression was assessed 48 hours after transduction by flow cytometry. Difference of GFP decrease between cells transduced by miR-150 or not is shown for each GFP reporter construction.

Supplementary reference.

1. Fang, Z. H. *et al.* miR-150 exerts antileukemia activity in vitro and in vivo through regulating genes in multiple pathways. *Cell Death Dis.* 7, e2371 (2016).
