# Supplementary material for: A miR-150/TET3 pathway regulates the generation of mouse and human non-classical monocyte subset
Source: Nat Commun. 2018 Dec 21;9:5455. doi: 10.1038/s41467-018-07801-x (PMC6303340; doi:10.1038/s41467-018-07801-x)
Supplement: Supplementary file 3 — Description of Additional Supplementary Files [file 41467_2018_7801_MOESM3_ESM.pdf]

Description of Additional Supplementary Files

File Name: Supplementary Table 2
Description: List of 367 RNA collected from hsa-miR-150 micro-RNA pull-down experiments in human CD14+ monocytes (N=4 independent experiments). Read count number are indicated for each of the four experiments. A threshold of more than 10 reads in at least 2 independent experiments was applied.

File Name: Supplementary Table 3
Description: List of the 2,176 differentially expressed genes between classical and nonclassical monocytes as determined by RNA-sequencing analyses of sorted cells. Adjusted P value < 0.01.

File Name: Supplementary Table 4
Description: Differentially expressed genes comparing classical or nonclassical monocytes in WT and Mir150-/- or WT and Tet3-/- mice as determined by RNA-sequencing analyses of sorted cells. Adjusted P value < 0.001.
