# Supplementary material for: A miR-150/TET3 pathway regulates the generation of mouse and human non-classical monocyte subset
Source: Nat Commun. 2018 Dec 21;9:5455. doi: 10.1038/s41467-018-07801-x (PMC6303340; doi:10.1038/s41467-018-07801-x)
Supplement: Supplementary file 7 — Reporting Summary [file 41467_2018_7801_MOESM7_ESM.pdf]

nature research

Corresponding author(s): Dorothee Selimoglu-Buet., or Eric Solary
email : dorothee.selimoglubuet@gustaveroussy.fr or eric.solary@gustaveroussy.fr

Reporting Summary

Nature Research wishes to improve the reproducibility of the work that we publish. This form provides structure for consistency and transparency in reporting. For further information on Nature Research policies, see Authors & Referees and the Editorial Policy Checklist.

Statistical parameters

When statistical analyses are reported, confirm that the following items are present in the relevant location (e.g. figure legend, table legend, main text, or Methods section).

n/a	Confirmed	
☐	☒	The exact sample size (*n*) for each experimental group/condition, given as a discrete number and unit of measurement
☐	☒	An indication of whether measurements were taken from distinct samples or whether the same sample was measured repeatedly
☐	☒	The statistical test(s) used AND whether they are one- or two-sided<br>*Only common tests should be described solely by name; describe more complex techniques in the Methods section.*
☐	☒	A description of all covariates tested
☐	☒	A description of any assumptions or corrections, such as tests of normality and adjustment for multiple comparisons
☐	☒	A full description of the statistics including central tendency (e.g. means) or other basic estimates (e.g. regression coefficient) AND variation (e.g. standard deviation) or associated estimates of uncertainty (e.g. confidence intervals)
☒	☐	For null hypothesis testing, the test statistic (e.g. *F*, *t*, *r*) with confidence intervals, effect sizes, degrees of freedom and *P* value noted<br>*Give P values as exact values whenever suitable.*
☒	☐	For Bayesian analysis, information on the choice of priors and Markov chain Monte Carlo settings
☒	☐	For hierarchical and complex designs, identification of the appropriate level for tests and full reporting of outcomes
☒	☐	Estimates of effect sizes (e.g. Cohen's *d*, Pearson's *r*), indicating how they were calculated
☐	☒	Clearly defined error bars<br>*State explicitly what error bars represent (e.g. SD, SE, CI)*

Our web collection on statistics for biologists may be useful.

Software and code

Policy information about availability of computer code

Data collection	FACS data collection was performed with BD FACSDIVA software (BD Biosciences)<br>Real time PCR 7500 software (life technologies)

Data analysis	FACS data analyzed with Kaluza Software (Beckman Coulter).<br>Statistical analysis and plotting Prism.<br>CRISPOR, ImageJ,<br>Integrative Genomics Viewer (IGV 2.1)<br>FastQC (v0.11.7), MultiQC (v1.5)<br>HOMER (v4.7.2), computeMatrix, plotHeatmap, bamcomverage from deeptools 3.1.2,<br>Trimmomatic (v0.32), Tophat2 (v2.0.14) / Bowtie2 (v2.1.0) , Bedtools (v2.17.0)<br>HTSeq (0.5.4p5) , DESeq2 (v1.10.1) package<br>Salmon (v0.10.2), pheatmap, an R package, Ward.D2 , Sleuth (v0.29.0), wasabi (v0.2).

For manuscripts utilizing custom algorithms or software that are central to the research but not yet described in published literature, software must be made available to editors/reviewers upon request. We strongly encourage code deposition in a community repository (e.g. GitHub). See the Nature Research guidelines for submitting code & software for further information.

Data

Policy information about availability of data

All manuscripts must include a data availability statement. This statement should provide the following information, where applicable:

- Accession codes, unique identifiers, or web links for publicly available datasets
- A list of figures that have associated raw data
- A description of any restrictions on data availability

Datasets are available in the ArrayExpress database at EMBL-EBI (www.ebi.ac.uk/arrayexpress). H3K4me3 CHIP-Seq comparing CMML and healthy donor monocytes profiles are referenced as E-MTAB-6689 and those comparing human Classical, Intermediate or Nonclassical monocytes profiles are referenced as E-MTAB-7290. RNA-seq comparing CMML and healthy donor monocytes expression profiles are referenced as E-MTAB-6712 and those RNA-seq comparing classical or nonclassical monocytes expression profiles between WT and MiR-150-/- or WT and Tet3-/- mice are referenced as E-MTAB-7291. GRO-cap analyses obtained in a human myeloid (K562) and a human lymphoid (GM12878) cell line presented Supplementary figure 7A were collected from www.ncbi.nlm.nih.gov/geo/ (GSE60456 accession). ChIP-seq data for H3K27me3, H3K4me1, H3K4me3 and H3K27ac obtained in human CD3+, CD19+, CD56+ and CD14+ cells presented Figure 5C were collected from www.roadmapepigenomics.org/.

Field-specific reporting

Please select the best fit for your research. If you are not sure, read the appropriate sections before making your selection.

☒ Life sciences ☐ Behavioural & social sciences ☐ Ecological, evolutionary & environmental sciences

For a reference copy of the document with all sections, see nature.com/authors/policies/ReportingSummary-flat.pdf

Life sciences study design

All studies must disclose on these points even when the disclosure is negative.

Sample size	Sample size was chosen based on material available ensuring that it will be appropriate for statistical analysis.<br>For all in vitro experiments, at least three biological replicates were used.<br>For in vivo experiments, at least five mice were used except for Tet3-/- model, three mice were used.<br>See figure legends.
Data exclusions	No data exclusion
Replication	All attempts at replication were succesfull
Randomization	Not relevant, when treatments were compared, cells were isolated from same donor or comparison between healthy donors and patients
Blinding	Experiments were neither randomised nor blinded

Reporting for specific materials, systems and methods

Materials & experimental systems

n/a	Involved in the study
☐	☒ Unique biological materials
☐	☒ Antibodies
☐	☒ Eukaryotic cell lines
☒	☐ Palaeontology
☐	☒ Animals and other organisms
☐	☒ Human research participants

Methods

n/a	Involved in the study
☐	☒ ChIP-seq
☐	☒ Flow cytometry
☒	☐ MRI-based neuroimaging

Unique biological materials

Policy information about availability of materials

Obtaining unique materials	All unique materials (plasmids) are available from the authors on demand.

Antibodies

Antibodies used	H3K4me3 antibody for Chip-seq (lot 12613005, n°39159) (Active Motif, La Hulpe, Belgium).<br>For immunoblot experiments, we used an anti-TET3 antibody from GeneTex Inc (Clinisciences, Nanterre, France), anti-HSC70 from Santa CruzBiotechnology antibodies (Clinisciences) and anti-ACTIN (A5441) from Sigma-Aldrich (Saint-Quentin Fallavier, France). Secondary antibodies were purchased at ThermoFisher Scientific (anti-rabbit, anti-mouse).<br>All FACS antibodies were purchased from BD biosciences (San Diego, USA), Biolegend (San Diego, USA), Beckman Coulter (Villepinte, France). Catalog numbers, suppliers, clone and fluorochrome are present in the antibodies supplemental table 4. See methods.
Validation	All western blots antibodies are commercially available and have been tested for the species used in this manuscript.<br>All FACS antibodies are commercially available and have been tested for the species used in this manuscript.

Eukaryotic cell lines

Policy information about cell lines

Cell line source(s)	The K562 cell line, originally established from the pleural effusion of a patient with chronic myeloid leukemia, and the U937 cell line, established from a histiocytic lymphoma, were purchased from ATCC.
Authentication	n.a.
Mycoplasma contamination	Cell lines were negative for mycoplasma contamination.
Commonly misidentified lines<br>(See ICLAC register)	n.a.

Animals and other organisms

Policy information about studies involving animals; ARRIVE guidelines recommended for reporting animal research

Laboratory animals	Wildtype CD45.1 and CD45.2, GFP and miR-150-/- mice were purchased from Jackson Laboratories (Charles River France, L'Arbresle, France).<br>Mice harboring Tet3 allele with the coding sequences of exon 11 flanked by two loxP were generated by the Plateforme Recombinaison homologue (Institut Cochin, Paris, France) and were intercrossed with mice expressing tamoxifen-inducible Cre (Cre-ERT) transgene under control of the Scl/Tal1 promoter/enhancer. To delete Tet3 floxed alleles, tamoxifen (Sigma) was solubilized at 20 mg/ml in sunflower oil (Sigma) and 8 mg tamoxifen were administrated to mice once per day for 2 days via oral gavage.<br>For monocyte quantification in mice cohorts, female and male were studied, for age, see legends.<br>For mice experiments (competition, transfer, cell autonomous...), female of 6-8 weeks were used.
Wild animals	n.a.
Field-collected samples	For competitive and rescue experiments, recipient mice were housed in a barrier facility under pathogen-free conditions after transplantation.

Human research participants

Policy information about studies involving human research participants

Population characteristics	For CMML patients, See Methods paragraph "healthy donor and patient samples" and supplemental table 1.<br>No data available for healthy donors
Recruitment	A learning cohort of CMML (Table S1) was made of patients enrolled between November 2008 and June 2009 in a previously reported phase 2 clinical trial described in Braun T et al. Blood 2011.<br>A validation cohort (Table S1) was made of 139 newly diagnosed CMML patients whose samples were collected with informed consent following the authorization provided by the ethical committee Ile-de-France 1 (DC-2014-2091).

ChIP-seq

Data deposition

☒ Confirm that both raw and final processed data have been deposited in a public database such as GEO.

☒ Confirm that you have deposited or provided access to graph files (e.g. BED files) for the called peaks.

Data access links<br>*May remain private before publication.*	Data availability. Datasets are available in the ArrayExpress database at EMBL-EBI (www.ebi.ac.uk/arrayexpress). H3K4me3 CHIP-Seq comparing CMML and healthy donor monocytes profiles are referenced as E-MTAB-6689 and those comparing human Classical, Intermediate or Nonclassical monocytes profiles are referenced as E-MTAB-7290. RNA-seq comparing CMML and healthy donor monocytes expression profiles are referenced as E-MTAB-6712 and those RNA-seq comparing classical or nonclassical monocytes expression profiles between WT and MiR-150-/- or WT and Tet3-/- mice are referenced as E-MTAB-7291.
Files in database submission	CTL1/input<br>CTL1/H3K4me3<br>CTL2/input<br>CTL2/H3K4me3<br>CTL3/input<br>CTL3/H3K4me3<br>CMML1/input<br>CMML1/H3K4me3<br>CMML2/input<br>CMML2/H3K4me3<br>CMML3/input<br>CMML3/H3K4me3<br>CMML4/input<br>CMML4/H3K4me3<br>CTL4 Classical/input<br>CTL4 CLassical/H3K4me3<br>CTL5 Classical/input<br>CTL5 CLassical/H3K4me3<br>CTL4 Intermediate/input<br>CTL4 Intermediate/H3K4me3<br>CTL5 Intermediate/input<br>CTL5 Intermediate/H3K4me3<br>CTL4 NonClassical/input<br>CTL4 NonCLassical/H3K4me3<br>CTL5 NonClassical/input<br>CTL5 NonCLassical/H3K4me3
Genome browser session<br>(e.g. UCSC)	n.a.

Methodology

Replicates	Figure 5C : 3 controls and 4 CMML<br>Figure 7 : 2 biological replicates (2 controls)
Sequencing depth	at least 20 millions reads per sample, see methods.
Antibodies	H3K4me3 antibody for Chip-seq (lot 12613005, n°39159) (Active Motif, La Hulpe, Belgium).
Peak calling parameters	Peak calling assessed using MACS 2.0 with a q-value cut-off of 0.05 for histone marks
Data quality	The quality of the sequences were first analyzed with FASTQC v0.11.2 and Trimmed with Trimmomatic v0.32 if necessary. Also to compute informative enrichment and quality measures of our CHIP-seq data, we used the R package phatompeakqualltools v1.10.1. With this package, we were able to represent the strand cross-correlation profiles which reflect the quality of the enrichment and predict the predominant fragment lenght. The NSC (Normalized Strand Cross-

	correlation coefficient) and RSC (Relative Strand Cross-correlation coefficient) values were calculated in the same time. These values allow us to know if our datasets had a low or high quality.<br>Then we calculated the PBC (PCR Bottleneck Coefficient) values that is a measure of library complexity.
Software	Reads were aligned into human genome hg19 with BWA aln (v0.7.5a) and peak calling assessed using MACS 2.0. Annotation and motif analyses have been done with HOMER (v4.7.2), with a p value of 0.01. Integrative Genomics Viewer (IGV 2.1) was used for representation.<br>Normalised bigwig files have been computed using bamcomverage from deeptools 3.1.2, with a bin size of 50, the number of read per bin normalized using RPKM methods, reads extension set to 250 bp, a smooth length windows of 150 bp. Reads with the same orientation and start have been considered only once. From normalized bigwig files, the distribution of signal for each mark and the corresponding heatmap have been computed using computeMatrix and plotHeatmap from deeptools 3.1.2, using TSS from Human genome annotation file (genecode.v19.annotation.gft) as the reference point.

Flow Cytometry

Plots

Confirm that:

☒ The axis labels state the marker and fluorochrome used (e.g. CD4-FITC).

☒ The axis scales are clearly visible. Include numbers along axes only for bottom left plot of group (a 'group' is an analysis of identical markers).

☒ All plots are contour plots with outliers or pseudocolor plots.

☒ A numerical value for number of cells or percentage (with statistics) is provided.

Methodology

Sample preparation	See methods, paragraph "Flow cytometry analysis"
Instrument	Cell fluorescence analyses were performed using a Fortessa (BD biosciences).<br>Samples were sorted using using Aria III, Aria-Fusion or Influx (BD Biosciences).
Software	Data collection was performed with BD FACSDIVA software (BD Biosciences) and analyzed with Kaluza Software (Beckman Coulter).
Cell population abundance	Post-purities were greater 95%
Gating strategy	For quantification of monocyte subsets in mice, gating strategy are described in supplemental figure 3. For apoptosis and progenitors quantification in mice, gating strategy are described supplemental figure 4. For monocyte transfer experiments, gating strategy is explained in supplemental figure 5bis . For monocyte subsets quantification in human cultures, gating strategy is explained in supplemental figure 5.

☒ Tick this box to confirm that a figure exemplifying the gating strategy is provided in the Supplementary Information.
